# Venom Peptides, Polyphenols and Alkaloids: Are They the Next Antidiabetics That Will Preserve β-Cell Mass and Function in Type 2 Diabetes?

**DOI:** 10.3390/cells12060940

**Published:** 2023-03-20

**Authors:** Michele Lodato, Valérie Plaisance, Valérie Pawlowski, Maxime Kwapich, Alexandre Barras, Emeline Buissart, Stéphane Dalle, Sabine Szunerits, Jérôme Vicogne, Rabah Boukherroub, Amar Abderrahmani

**Affiliations:** 1 University Lille, CNRS, Centrale Lille, University Polytechnique Hauts-de-France, UMR 8520, IEMN, F-59000 Lille, France; 2 Service de Diabétologie et d’Endocrinologie, CH Dunkerque, 59385 Dunkirk, France; 3 Institut de Génomique Fonctionnelle, Université de Montpellier, CNRS, INSERM, 34094 Montpellier, France; 4 University Lille, CNRS, Inserm, CHU Lille, Institut Pasteur de Lille, U1019-UMR 9017-CIIL-Center for Infection and Immunity of Lille, F-59000 Lille, France

**Keywords:** diabetes, insulin secretagogues, pancreatic beta cell, venom, polyphenols, alkaloids, peptides

## Abstract

Improvement of insulin secretion by pancreatic β-cells and preservation of their mass are the current challenges that future antidiabetic drugs should meet for achieving efficient and long-term glycemic control in patients with type 2 diabetes (T2D). The successful development of glucagon-like peptide 1 (GLP-1) analogues, derived from the saliva of a lizard from the *Helodermatidae* family, has provided the proof of concept that antidiabetic drugs directly targeting pancreatic β-cells can emerge from venomous animals. The literature reporting on the antidiabetic effects of medicinal plants suggests that they contain some promising active substances such as polyphenols and alkaloids, which could be active as insulin secretagogues and β-cell protectors. In this review, we discuss the potential of several polyphenols, alkaloids and venom peptides from snake, frogs, scorpions and cone snails. These molecules could contribute to the development of new efficient antidiabetic medicines targeting β-cells, which would tackle the progression of the disease.

## 1. Introduction

Humanity has always been inspired and guided by plants and animals for its healthcare [1], as cited by Hippocrates: “*Nature itself is the best physician*”. Plants have been able to offer efficient analgesics (morphine and codeine), anti-cancer (taxol), antiparasite (artemisinin) as well as anti-inflammatory (salicylic acid) drugs. In addition to bacteria and fungi, many analgesics, vaccines (hepatitis A, influenza), inflammation modulators and anti-venom drugs are from the animal origin [2]. Antidiabetic drugs have also been supplied from plants and animals. For example, dog insulin enabled Sir Frederik Banting to reveal the therapeutic activity of this hormone, and to treat the first patient with type 1 diabetes [3]. Before genetic engineering was possible, insulin, used to treat millions of patients, was from bovine and pig origin. For patients with type 2 diabetes (T2D), the most prominent type of diabetes with 90% of all diagnosed cases, several current antidiabetic drugs are from plant and animal origins: metformin, sodium glucose co-transporter type 2 inhibitors (SGLT2is) and glucagon-like peptide 1 receptor agonists (GLP-1RAs). Metformin is currently the most popular antidiabetic drug and the first line of medication in T2D. Metformin is a biguanide derivative, whose antidiabetic activity was originally described in the Middle Age with the use of the *Galega officinalis*, also known as “French lilac”, plant [4]. Metformin acts as an insulin sensitizer, which together with lifestyle changes, improves glucose uptake of patients, and thereby reduces hyperglycemia [4]. The antidiabetic effect mostly relies on the inhibition of hepatic gluconeogenesis [5,6]. With metformin, the SGLT2i antidiabetic class of T2D is becoming very popular [7]. Besides their glucose-lowering effect, SGLT2is reduce the risk of cardiovascular diseases and hospitalization caused by heart failure [8,9]. SGLT2is lower glucose blood levels by reducing renal glucose reabsorption and by promoting urinary glucose excretion [7]. Historically, SGLT2is originate from phlorizin, a naturally occurring glucoside found in various plants, such as the root bark of apple and other fruit trees [10]. While phlorizin was initially used for treating fever, infectious diseases and malaria, the substance could lead to glucosuria and polyuria [11]. Because phlorizin is poorly absorbed into the gastrointestinal tract and acts in other tissues by inhibiting SGLT1 (primarily found in the gastrointestinal tract), the molecule has never been used as a medication for the treatment of T2D. To circumvent these concerns, analogs of phlorizin have been developed. Another drug used for the treatment of T2D is GLP-1RA which improves insulin secretion in patients. Indeed, T2D develops when insulin secretion from pancreatic β-cells of the islet of Langerhans, the only cells in the body specialized in the production of insulin, is insufficient for coping insulin resistance [12]. GLP-1RA alleviates hyperglycemia by potentiating nutrient-induced insulin secretion [13]. Therefore, GLP-1RAs are of high value as they do not cause hypoglycemia. In addition, they are considered as very promising since they are the only antidiabetics that could slow and/or prevent the degradation of β-cell mass of patients, as revealed by preclinical studies [14]. In fact, β-cell mass in a T2D patient is only 40–60% of that of a body mass index-matched non-diabetic person [15,16]. Progressive reduction of β-cell mass contributes to the poor glycemic control of patients over time. This reduction is suggested to degenerate, as the remaining β-cells are functioning at very likely only half their capacity [17]. The first GLP-1RA was originally discovered by an American team through the search for molecules from arthropod and reptile venoms that activate G-protein coupled receptors (GPCRs) involved in pancreatic amylase secretion. The most potent molecule came from the venom of the Gila monster (*Heloderma suspectum*), in which resides the GLP-1RA Exendin 4 [18,19]. The half-life of the GLP-1RA has been extended by chemical modification, leading to exenatide and many other derived drugs so far [20]. Unfortunately, GLP-1RAs are heterogenous in terms of efficiency for achieving short- and mid-time glycemic control [21]. Their efficiency can even be reduced over time [22,23]. In addition, some patients are non-respondent to GLP-1RAs [24]. A randomized controlled trial, performed in a small cohort of 40 subjects with early T2D, were treated for 6 months with the GLP-1RA exenatide, but it had no effect on β-cell mass [25,26].

Similarly, the sulfonylureas (SUs) and glinides, two popular classes of oral antidiabetics which directly enhance insulin secretion, are unable to achieve long-term glycemic control, and thereby cannot be used as therapeutic alternative [27,28]. The concern is that these drugs do not preserve functional β-cell mass, which continues to deteriorate over time, worsening insulin deficiency. SUs can even participate in the functional β-cell mass demise by accelerating β-cell apoptosis and β-cell exhaustion or desensitization [29,30]. In addition, SUs and glinides stimulate insulin secretion in the absence of glucose or food uptake, which can cause hypoglycemia, a major side effect that can limit their use for some patients. Moreover, weight gain, nausea, erythema multiforme, exfoliative dermatitis and also, more rarely, photosensitivity are some of the secondary effects of these drugs [31,32]. Occasionally, they can cause also cardiac dysfunction, hyponatremia and abnormalities in liver function [33].

Therefore, it is urgent to develop insulin secretagogue drugs with long duration efficiencies that are capable to preserve functional β-cell mass by protecting them from death caused by the diabetogenic environment (cytokines, chronic hyperglycemia, chronic hyperlipidemia and amyloid deposits). To this end, it is essential that future drugs not only target the key cellular mechanisms that stimulate insulin secretion, but also promote β-cell survival in this detrimental environment. With approximately 8.7 million plant and animal species worldwide, including 6.5 million species living on earth and 2.2 million in the seas [34], it is highly possible to tackle this medical challenge. Indeed, there are accumulating data evidencing that animal venom peptides and plant substances including polyphenols and alkaloids are potential candidates. This review reports these molecules and provides an original and consistent presentation of their potential for targeting the mechanisms of insulin secretion and β-cell protection, the expected requirement of future antidiabetics.

## 2. Peptides and Substances Stimulating Insulin Secretion

### 2.1. Key Pathways Regulating Glucose-Induced Insulin Secretion

**Figure 1:**
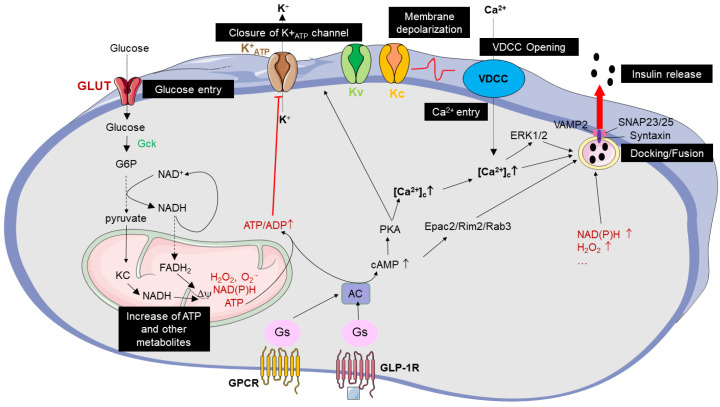
Triggering and amplifying pathways coupling glucose to insulin secretion. Glucose enters via glucose transporters (GLUTs) and increases the ATP/ADP ratio by glycolytic and TCA metabolism. Then, ATP promotes the closure of K^+^ channels, which stops the K^+^ efflux. This leads to membrane depolarization and the opening of voltage-dependent calcium channels (VDCCs). The entry of Ca^+^ could activate the mitogen-activated kinases ERK/2, which together fosters the fusion of insulin-containing granules with the plasma membrane, and finally the release of insulin into the extracellular compartment. The triggering pathway is followed by the amplifying pathways which involve GLP-1R and other G_s_-protein coupled receptors (GPCRs), and several metabolites including NADH/NADPH, radical species, H_2_O_2_, and lipidic metabolites. All of these can promote the granule docking, fusion and finally, insulin exocytosis. Granule fusion entails the pairing of the (v)-SNARE (VAMP2) and t-SNAREs (SNAP-25/23 and Syntaxin) proteins, forming a binary cognate target membrane receptor complex. After insulin release, the membrane repolarization involves Kv and Kc channels. GCK: glucokinase, Kv: voltage-dependent potassium channel, Kc: calcium-activated potassium channel, G6P: glucose-6 phosphate, AC: adenylate cyclase, PKA: Protein Kinase A, ERK1/2: extracellular signal-regulated kinases 1/2. VAMP2: vesicle-associated membrane protein 2, SNAP23/25: synaptosome-associated proteins 23/25 kDa.

Under physiological conditions, in the presence of non-stimulatory concentrations of glucose, low levels of insulin are secreted by β-cells. Basal insulin secretion results from the low rate of glucose metabolism, leading the opening of ATP-dependent potassium channels (K^+^_ATP_ channels). The potassium efflux counteracts depolarizing currents which thereby maintains the membrane’s steady-state potential at more negative values and the closure of the voltage-dependent Ca^2+^ channels. When the concentration of glucose increases, it enters into the cell and its metabolization through the glycolysis pathway and tricarboxylic acid cycle (TCA) is accelerated (Figure 1). This results in elevated mitochondrial ATP generation and a decrease in ADP concentration, which induces the closure of K^+^_ATP_ channels [35]. The closure of K^+^_ATP_, induced by higher a ATP/ADP ratio, prevents the K^+^ efflux and thereby causes membrane depolarization. Subsequently, the membrane depolarization leads to the opening of voltage-dependent calcium channels (VDCCs) and the influx of Ca^2+^. Finally, the rise of intracellular Ca^2+^ accounts for the insulin export through a soluble N-ethylmaleimide-sensitive factor attachment protein receptor-mediated (SNAREs) fusion of a readily releasable pool of insulin-containing vesicles with the plasma membrane [36,37,38]. This triggering mechanism involving K^+^_ATP_ is responsible for the first phase of the insulin secretory response. This phase occurs during the first 5–10 min. The second phase, termed as the amplifying pathway, is more sustained and is achieved over a period of 30–60 min. This second phase relies on a K^+^_ATP_-independent mechanism [39]. This mechanism involves several metabolites including TCA intermediates, such as NADPH and NADH, and associated products (anaplerosis), such as glutamate, malonyl-CoA, phospholipase C/protein kinase C (PKC) signaling, alterations in intracellular levels of lipids and/or elevation in cAMP levels, together enhancing cytosolic Ca^2+^ concentrations and insulin exocytosis (Figure 1).

### 2.2. Peptides from Animal Venoms That Act as Insulin Secretagogues

Within the animal kingdom, several strategies are used for defense and hunting. One consists of using poisons and venoms to subdue and/or kill prey or predators. Unlike poisons that induce their toxicity by ingestion or external contact, venoms have to be parenterally administrated via specialized apparatus (e.g., fangs, stingers, teeth, nematocysts). Some animals including snake, lizards, frogs, spiders, scorpions and cone snails, for example, produce and secrete venoms. Venoms contain a mixture of substances mainly enriched with proteins called “toxins”. In fact, toxins include enzymes (e.g., oxidases, hydrolases, proteases and phospholipases), non-enzymatic proteins (e.g., disintegrins) and peptides. Many of venom peptides modulate ion channels and receptors in a broad variety of species including humans [40]. Thanks to their mode of action, toxins from venoms are used in a wide-range of pharmaceutical and cosmeceutical activities [41]. Indeed, several drugs based on peptide toxins, including captopril (hypertension), ziconotide (chronic pain), eptifibatide (cardiovascular diseases), lepirudin (thrombosis, stroke) and cobratoxin (pain), are currently in clinical use [42]. Venoms can contain peptides that stimulate the production of hormones and growth factors, as illustrated by the venom from the snake *Bothrops jararaca* [43]. This venom contains prothrombin and factor X activators that can elicit the generation of hepatocyte growth factor/scatter factor (HGF/SF), a regenerative growth factor that is considered a therapeutic target in T2D [44]. Finally, some peptides from venom can mimic human hormones and thereby can be potentially used as medicines. This is illustrated by Exendin-4 which has led to the development of new generations of GLP-1RAs with longer half-lives [45]. The Exendin-4 story has been pivotal for supporting the idea that other peptides from venoms can be considered for developing antidiabetic medicines. Two decades of research have enabled the identification of other peptides capable of triggering insulin secretion. Some of these peptides are GLP-1RA, K^+^_ATP_ blockers or modulators of major channels regulating the triggering and/or amplifying pathways of glucose-induced insulin secretion (GSIS).

#### 2.2.1. Venom Peptides as New GLP-1RAs

Venom peptides target a wide variety of membrane-bound protein channels and receptors. GLP-1RA, that elevates cAMP levels by activating the G-protein coupled receptor (GPCR), supports the concept that molecules capable of modifying the β-cell membrane depolarization and/or modulating the key intracellular partners of the triggering and amplifying pathways are good insulin secretagogue candidates [46,47]. The discovery of Exendin-4 in the venomous saliva of the Gila monster and the multiple health benefits of GLP-1RAs [13], beyond lowering plasma glucose, have opened up new avenues of research for analogues from other animal species. The 13-amino-acid peptide (RK-13) isolated from the skin of *Agalychnis calcarifer* frogs might act as a GLP-1RA, although the binding to GLP-1R and the downstream receptor signaling have not yet been directly demonstrated [48]. Currently, efforts are focused on the identification of analogues with more selective therapeutic effects than those of Exendin-4 and native GLP-1. In particular, GLP-1 analogues, also called GLP-1 receptor-biased agonists (GLP-1RBAs), are expected to be the future drugs of this class. While GLP-1RBAs act through the same GLP-1 receptor, it improves the durability of the effect on insulin secretion [49]. Two GLP-1RBAs have been discovered in venoms of platypuses (*Ornithorhynchus anatinus*) and short-beaked echidnas (*Tachyglossus aculeatus*), two mammals of the monotreme order living in Australia and New Guinea [50]. The two GLP-1RBAs are structurally analogous to Exendin-4, although they differ by 12 amino acids in their sequence [50]. The affinity of both peptides for the human GLP-1 receptor is lower than native GLP-1 [50]. Nonetheless, these novel analogues stimulate insulin secretion in response to glucose through the preferential activation of one of the MAPK signaling pathways (ERK1/2) [50]. In addition, both peptides are more resistant to digestion by dipeptidyl peptidase-4 (DPP-4) than Exendin-4 [50], thus confirming the feasibility for the development of new GLP-1 analogues with longer-lasting and more specific effects.

#### 2.2.2. K^+^_ATP_ Channel Inhibitor Peptides from Venom

The closure and opening of K^+^_ATP_ channel are pivotal for controlling insulin secretion. This channel consists of four sulfonylurea receptors (SURs) surrounding four pore-forming subunits named Kir6.1 or Kir6.2 [51]. Channel activity involves the interaction of ATP or ADP with the two nucleotide-binding sites of SUR. When glucose concentration rises in plasma, it is sensed by the β-cell thanks to its facilitated passage into cytoplasm via the low K_m_ glucose transporters (GLUT2). The increase in glucose metabolism promotes ATP synthesis. ATP binding to SUR1 causes K^+^_ATP_ channel closure, inhibition of the K^+^ efflux, β-cell membrane depolarization, calcium influx and finally insulin secretion [51]. SUs can also directly stimulate insulin exocytosis by penetrating into β-cells and triggering its secretory machinery [52]. SUs cause a maximum channel blocking of ~50–80% [53], thereby stimulating insulin secretion. The first members of SUs for treating T2D were tolbutamide, chlorpropamide, acetohexamide and tolazamide [54]. Second-generation and third-generation SUs were developed later including glyburide, glipizide and glimepiride [55,56]. Another class of oral antidiabetic targeting K^+^_ATP_ channels is the glinides [57]. Like SUs, all glinides promote closure of the K^+^_ATP_ channels. However, unlike SUs, glinides bind directly to the Kir6.1 subunit [57]. Glinides, including repaglinide (RPG), meglitinide, mitiglinide and nateglinide, are widely prescribed, because of their good safety and efficacy for controlling postprandial blood glucose by stimulating insulin secretion. However, glinides can also provoke hypoglycemia as they elicit insulin secretion in a glucose-independent manner. Nonetheless, the release of SU and glinides has been instrumental for serving as models for identifying other therapeutic peptides capable of blocking K^+^_ATP_ channels by activating the Kir6.1 subunit or SUR. At the present time, several insulin secretagogue peptides from animal venom inducing the closure of the K^+^_ATP_ channel have been identified (Table 1). However, unlike SUs and glinides, for most peptides, their effects on the K^+^_ATP_ channels seem to be indirect. This is the case for mastoparan, tigerinin and secretory phospholipase 2 [58,59,60]. Only the protein toxin dubbed SpTx1, isolated from the venom of desert centipede *Scolopendra polymorpha*, has been shown to directly interact with the K^+^_ATP_ channel [61]. In addition, SpTx1 inhibits K^+^_ATP_ channels by blocking the ion-conduction pore [62]. However, the usage of this peptide as K^+^_ATP_ inhibitor should raise the question of their safety. Besides the risk of hypoglycemia, the same drawbacks as SUs and glinides, the K^+^_ATP_ channel inhibitor peptides might affect cardiomyocytes, where the channel is abundantly expressed. This hypothesis is supported by SUs, which might increase the risk of cardiovascular events by targeting the K^+^_ATP_ channel [63]. The search for K^+^_ATP_ channel inhibitor peptides as insulin secretagogues definitely requires further investigations of their effects in heart function.

#### 2.2.3. Venom Peptides Inhibiting Voltage-Dependent (Kv) and Calcium-Activated (Kc) Potassium Channels

Another strategy for stimulating GSIS is to inhibit β-cell membrane repolarization controlled by voltage-dependent (Kv) and calcium-activated (Kc) high conductance K^+^ channels. Kv and Kc open upon membrane depolarization and mediate outwardly rectifying K^+^ currents, which act to repolarize action potentials [69]. Kv and Kc channels are a homo- or heterotetrameric complex of α-subunits of the same family. Kv2.1 is the major β-cell Kv channel isoform. Some peptides from venoms of striated cones, tarantulas and scorpions have been identified to be capable of inhibiting Kv channel activity (Table 2). These peptides are supposed to maintain the β-cell in a depolarized state, which would prolong insulin secretion only in the presence of glucose. However, Kv and Kc are also expressed in bladder and other excitable cells of the neuronal and cardiovascular systems [70,71]. Therefore, further investigations are required for controlling the side effect of peptides in clinical applications.

#### 2.2.4. Peptides That Stimulate Insulin Secretion in a Not Yet Identified Mechanisms

A large number of toxins are ion channel modulators that can inhibit or activate metabolic enzymes. If the venom peptides act as ligands of ion channels, they also possess other favorable characteristic features such as small size with high stability, cationicity and hydrophobicity. For example, cationicity promotes peptide–cell membrane interactions and subsequent internalization. These physicochemical characteristics provide peptides some key advantages as antimicrobial agents [76,77]. Some of these peptides have led to Captopril, an angiotensin converting enzyme inhibitor, which is derived from the venom of the *Bothrops jararaca* viper [78]. Captopril is prescribed for the treatment of hypertension, diabetic nephropathy and heart failure [79]. Several cationic venom peptides with insulin secretagogue activity have been identified (Table 3). However, the mechanisms through which they stimulate insulin secretion are not elucidated. Thanks to their cationicity, it is suggested that these peptides penetrate membranes and enter into cells to stimulate insulin secretion via mechanisms that do not require K^+^_ATP_. These peptides that can enter into cells include bombesin [80]; crotamine [81] members from *Pipidae* and *Ranidae* families isolated from the skin of amphibians [82]; Brevinin-2-related peptide (B2RP), a peptide of the northern frog (*Lithobates septentrionalis*); Alyteserin-2a of the midwife toad (*Alytes obstetricans*); Hymenochirin-1b of the African dwarf frog (*Hymenochirus boettgeri*); Magainin-AM1 and AM2 of *xenopus amieti*; and Esculentin-2Cha of the Chiricahua leopard frog (*Lithobates chiricahuensis*). The peptides enter into the cells, depolarize the β-cell membrane and stimulate insulin secretion [83]. These peptides could pave the way for the development of a new class of antidiabetic drugs, although they may also lead to hypoglycemia.

### 2.3. Polyphenols and Alkaloids from Plants Stimulating Insulin Secretion

**Figure 2:**
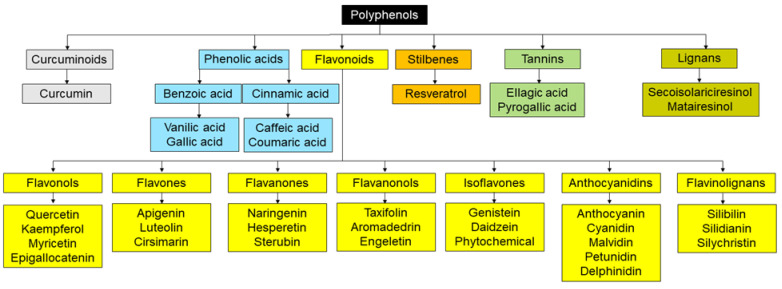
Classification of Polyphenols.

Medicinal plants have been a major focus of research due to the presence of bioactive compounds that may provide the foundation for drug design. The World Health Organization lists almost 21,000 plants used for medicinal purposes worldwide [110]. Bioactive substances include mostly polyphenols and alkaloids. Polyphenols are secondary polyhydroxy phytochemicals metabolites resulting from the shikimic acid and phenylpropanoid pathways of plants. These metabolites mediate plant defenses against pathogenic aggression and ultraviolet radiation [111]. They are also key for plant adaptation against stressful environments and cues [112]. These phenolic substances share a common phenolic ring structure, with one or more phenolic rings linked to more than one hydroxyl group [113]. Polyphenols are classified into several subgroups (Figure 2) with flavonoids being the largest one [114].

**Figure 3:**
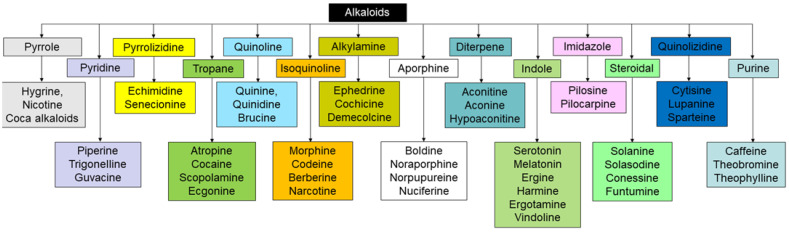
Classification of Alkaloids.

Alkaloids are nitrogenous compounds derived from the metabolism of amino acids, such as tyrosine, lysine, ornithine, phenylalanine and tryptophan. They contain at least one nitrogen atom in a heterocyclic ring. In addition, most alkaloids contain oxygen. The term alkaloid refers to the basic (alkaline) nature of the structure. There are several groups of alkaloids (Figure 3), which are mainly found as salts or as N oxides in seed-bearing plants, in berries, bark, fruits, roots and leaves. They are also found in marine algae [115] and in the skin of amphibians along with other toxins [116]. Among more than twenty thousand alkaloids, several dozen are currently used as medical drugs as exemplified by morphine and codeine [117]. An application of alkaloids and polyphenols for the treatment of diabetes is possible. The literature reports a plethora of studies confirming some direct effects of polyphenols and alkaloids on insulin secretion. Some of them inhibit insulin secretion, as exemplified by colchicine [118], scopolamine [119,120], melatonin [121,122], atropine [123], cystisine [124] and serotonine [125]. For some others, there are still some debates and conflicting results. For example, according to the concentration, nicotine [124,126,127], some quinoline members (e.g., quinine and quinidine) [128,129] and some isoquinoline members (e.g., berberine) [130,131,132] can either stimulate or inhibit insulin secretion. Nonetheless, numerous polyphenols and alkaloids have been identified as direct insulin secretagogues. However, the molecular mechanisms of their effects are different. While some polyphenols, such as resveratrol, cyanidin and rutin, stimulate insulin secretion via an increase in glucose metabolism or a direct augmentation of Ca^2+^ influx [133,134,135], other polyphenol substances and alkaloids enhance insulin secretion via other pathways. Some molecules can directly trigger the closure of K^+^_ATP_ (Table 4), although it is unclear if they directly close the K^+^_ATP_ channels by binding to SUR or Kir6.2 and/or indirectly induce the closure through an increase in ATP production. Other molecules promote the rise of cAMP levels similar to GLP-1RAs (Table 5). Unlike myricetin, the mechanism through which compounds such as vanillic acid stimulate the rise of cAMP is not well understood. Curcumin could also induce the rise of cAMP via the inhibition of phosphodiesterase activity [136], whereas genistein and daidzein seem to directly stimulate adenylate cyclase activity similar to forskolin [137]. Quercetin may activate β-adrenergic receptors [138], whereas the alkaloid morphine could involve opioid receptors [139].

## 3. Venom Peptides, Polyphenols and Alkaloids Protecting β-Cells against Death Induced by Diabetogenic Environments

### 3.1. Preserving β-Cell Mass in T2D by Antagonizing ER Stress, Oxidative Stress and Autophagy as the Paradigm for Achieving Long-Term Glycemic Control in T2D

In T2D, β-cell death is the leading cause in the reduction of β-cell mass [16,162] although the increase of β-cell senescence [163] and dedifferentiation [164] are also involved. Nowadays, there is evidence that β-cell death results from activation of several pathways including Endoplasmic Reticulum (ER) stress [165], oxidative stress (OS) [166] and autophagy [167,168], which intersect with one another. Amyloid deposits, pro-inflammatory cytokines, hyperlipidemia (cholesterol and saturated fatty acids) and hyperglycemia are, individually and in combination, involved in the induction of ER stress, OS and autophagy [169,170]. When compared to SUs, glinides and gliptines, the GLP-1RAs are the only antidiabetic drugs that can antagonize the deleterious effects of ER stress [14], oxidative stress [171] and autophagy in β-cells [172]. Although the preclinical data are exciting, in clinical setting, the long-term benefits of some GLP-1RAs are debated as many patients are non-responders to GLP-1RAs and switch to insulin therapy [173]. One explanation is that the expression of GLP-1 receptor (GLP-1R) is decreased in patients with T2D [174,175]. The insufficient GLP-1R levels in β-cells could reduce the biological effects of GLP-1RAs and thereby limit their use in some patients. Therefore, there is an urgent need that the next generation of antidiabetics that targets β-cells not only improve insulin secretion, but also protect them against death caused by stress-induced pathways.

### 3.2. Survival Proteins of β-Cells Revealed by GLP-1RAs

Intensive studies have tried to unravel the mechanism through which GLP-1RAs antagonize the deleterious effects of ER stress [14], oxidative stress [171] and autophagy [172]. These mechanisms have been instrumental for identifying the key targets required for β-cell protection. These proteins are listed in the Table 6 and are considered as key players in the β-cell protection elicited by GLP-1RAs when they meet the following criteria: (1) they are activated and/or their expression induced by GLP-1RAs in β-cells and (2) their inhibition and/or suppression attenuate the protective effect of GLP-1RAs on cell death induced by pro-apoptotic stressors. All these proteins belong to the GLP-1RA signaling cascade and are therefore connected with each other, as exemplified by the IB1/JIP1/JNK3 pathway [176]. Therefore, targeting these proteins using GLP-1RAs represents a relevant therapeutic strategy for improving β-cell mass in T2D.

### 3.3. Peptides from Venoms That Protect β-Cells against Death by Targeting β-Cell Survival Proteins

Despite the identification of venom peptides that can act as insulin secretagogues, there are few in vitro and preclinical studies stating a direct protective effect of these peptides in β-cell death. In addition, most studies did not validate the direct role of the peptides in β-cell viability and/or β-cell mass, although plasma glucose, insulin level and blood biomarkers have been investigated [82]. In fact, only considering the in vitro and in vivo studies that have directly investigated β-cell viability, very few peptides from venoms have been tested among those exhibiting an insulin secretagogue activity. Temporins A and F protect BRIN-BD11 cells against death [206]. However, the mechanism through which temporins trigger β-cell protection has not been elucidated thus far. A protective role of esculentin-2Cha and PGLa-AM1 in an in vitro model of β-cells, possibly via the induction of PDX1, has been described [106,207]. Nonetheless, these results still need to be confirmed in human islets and islets of animal models of T2D.

### 3.4. Polyphenols and Alkaloids That Protect β-Cells against Death by Targeting the β-Cell Survival Proteins

Unlike the peptides from venom, the literature is more substantial regarding studies investigating the protective effect of polyphenols and alkaloids on β-cell death. Dozens of these plant substances (Table 7), mostly polyphenols, have been directly tested for their capacity to counteract the toxicity induced by diabetogenic factors and exploring the underlying mechanisms. Besides stimulating insulin secretion (see Section 2.3), curcumin, cyanidin, kaempferol, quercetin, myricetin, genistein, silibilin and resveratrol, seem to directly protect β-cells similar to GLP-1RAs. It is noteworthy that the protective effect of flavonol, curcuminoid, flavone, isoflavone, flavinolignan and stilbenes on β-cells could also rely on their phytoestrogen activity in a mechanism involving estrogen receptors (ER). Indeed, as phytoestrogens, the members of the six polyphenol subclasses can bind to both types of ERα receptors and ERβ receptors, mimicking the effect of estradiol [208,209,210]. Estradiol prevents β-cells death induced by OS [211]. However, the estradiol-mimicking effect of these polyphenols in β-cell protection remains to be confirmed. In addition, at the present time, evidence supporting the role of polyphenols and alkaloids for reducing hyperglycemia and improving β-cell mass and function in human are missing. Resveratrol fails to restore glycemia and to improve insulin secretion in a clinical trial of diet-controlled patients with T2D treated for 5 weeks with stilbene [212]. In other clinical trials, although promising, the data are incomplete as only mixtures of polyphenol-enriched extracts were used. Using either polyphenol-rich drinks [213] or polyphenol-enriched plant extracts, the clinical studies showed an improvement in fasting and/or postprandial glycemia in healthy individuals or people with metabolic syndrome or with T2D [214,215,216]. In a 3-month period trial, curcumin administrated in patients with T2D improved glycemia and plasma insulin levels [217]. Among alkaloids, berberine is one of the most intensively studied [218]. Besides the improvement of insulin sensitivity, hepatic lipid metabolism and adipose fibrosis, this isoquinoline might also alleviate hyperglycemia by protecting β-cells against death induced by lipotoxicity in a mechanism involving SIRT1 [219]. Although berberine seems convincing as a promising antidiabetic, the temporary adverse gastrointestinal events observed in one third of patients treated with the alkaloid in a trial of 59 patients [220], has restrained the medical community from using it for clinical purposes. This worry is further supported by the inconsistent bioavailability of berberine after oral ingestion, as shown by a randomized, double-blind, placebo-controlled investigation [221]. In addition, as mentioned above (see Section 2.3), there are conflicting results of the effect of berberine on insulin secretion [130,132]. Therefore, future experimental and clinical studies are required for confirming the effectiveness of berberine on insulin secretion and its long-term safety in a consistent cohort of patients.

## 4. Conclusions

The fight against diabetes epidemic worldwide requires efficient drugs that not only improve β-cell function, but also preserve their mass [247]. This review underlines that only polyphenols and one alkaloid, berberine, have been clearly studied as insulin secretagogues and β-cell protectors, whereas the effects of animal venom peptides in the preservation of β-cell are largely under investigated. Flavonols, curcuminoids, flavones, isoflavones, flavinolignans and stilbenes can be considered as the most promising drugs, as exemplified by curcumin, resveratrol, silibilin, genistein, myricetin, quercetin, EGCG and apigenin. In addition, as phytoestrogens, all these polyphenols could provide several additional benefits for patients. Phytoestrogens are reported to lower the risk of menopausal symptoms, cardiovascular diseases, brain function disorders and several cancers such as breast, bowel, uterine and prostate cancers [248]. Nonetheless, the interest in these polyphenols for clinical use will only be approved if future studies at least confirm their safety in terms of infertility risks and increased risks of cancer in estrogen-sensitive organs. The careful attention to their safety is further supported by the fact that polyphenols also target a large number of receptors and non-receptor tyrosine kinases and serine–threonine kinases, which play key pleiotropic roles in cellular signaling and physiology [249,250]. Besides their safety, there is still a need for additional preclinical studies to confirming their bioavailability, pharmacokinetics and efficiency upon delivery by an oral route. Successful delivery of polyphenols and alkaloids via this route could offer some metabolic advantages as these compounds might directly stimulate GLP-1 secretion in the gut. Curcumin, delphinidin, EGCG and genistein, for example, are able to stimulate GLP-1 secretion in in vitro and rodent diabetic models [251]. In the gut, the polyphenols could also be beneficial by acting as prebiotics for stimulating the production of *Akkermansia muciniphila*, a bacteria that improves the glucose metabolism of patients with T2D [252], as shown by a previous study using polyphenol-rich extracts [253]. However, oral administration of polyphenols is challenging as it compromises the stability of the substances in the gastrointestinal tract and their proper absorption. Polyphenols are not well assimilated by the gut if they are delivered as glycosides, esters or polymers [254]. In addition, they can be modified by intestinal bacteria and trigger some side effects including nausea, headache and nasopharyngitis, even though the available data from clinical studies are rather optimistic, showing that polyphenols are overall safe and cause marginal side effects [255].

Encapsulation of peptides, polyphenols and alkaloids into biocompatible nanoparticles/nanocapsules will increase their apparent solubility, bioavailability and intestinal permeability and reduce their side effects [256,257,258]. Optimal formulation of these substances with nanoparticles and their preclinical validation are required before proceeding to clinical trials. The latter should be randomized and performed in large cohorts of patients receiving the therapeutic substances for periods of over 6 months. In addition, for validating the properties of the substances on β-cell mass and function, it is essential that trials include the current methods for monitoring β-cell function and mass such as arginine-induced insulin secretion, mixed meal tolerance tests, oral glucose tolerance tests and/or intravenous glucose tolerance tests [259].

## Figures and Tables

**Table 1:** K^+^_ATP_ inhibitor peptides that stimulate insulin secretion.

Compounds	Specie	*In Vitro* Models	*In Vivo* Models	Reference(s)
**SpTx1**	*Scolopendra polymorpha*	Isolated mouse islets	Wild type mice	[62]
**Mastoparan**	*Vespula lewisii*	Rat RINm5F, hamster HIT-T15, mouse αTC3 cells, rat INS-1 cells isolated rat and human islets	ND	[58,64,65,66]
**Secretory** **phospholipase 2**	*Naja mossambica*	Isolated mouse islets and single β-cells	ND	[60]
**Tigerinin-1R and analogs**	Indian frog *Hoplobatrachus tigerinus*	BRIN-BD11 cells	HFD-induced Swiss obese mice	[59,67,68]

ND: Not Done, High Fat Diet: HFD.

**Table 2:** Calcium-dependent potassium (K_C_) or voltage-dependent potassium channels (K_V_) inhibitor peptides that stimulate insulin secretion.

Compounds	Specie	Class of the Active Substance	*In Vitro* Models	*In Vivo* Models	Reference(s)
**Conkunitzin-S1**	Striated Cone (*Conus striatus*)	Inhibitor of K_V1.7_	Isolated rat islets	ND	[72]
**Guangxitoxin-1 (GxTX-1)**	Chinese Fawn Tarentula (*Chilobrachys Guangxiensis)*	Inhibitor of K_V2.1_ and K_V2.2_	Isolated mouse islets	ND	[67]
**Hanatoxin (HaTX)**	Chilean Rose Tarentula (*Grammostola rosea*)	Inhibitor of K_V2.1_	Isolated human islets	ND	[73]
**Iberiotoxin**	Eastern Indian Scorpion *(Hottentotta tamulus)*	Inhibitor of K_C_	Isolated human islets, mouse MIN6 cells	ND	[74,75]

ND: Not Done.

**Table 3:** Peptides stimulating insulin secretion with unknown mechanisms.

Compounds	Specie	*In Vitro* Models	*In Vivo* Models	Reference(s)
**Agelaia MP-I (AMP-I)**	Vespid wasp *(Agelaia pallipes pallipes)*	Isolated mouse islets	ND	[84]
**Alyteserin-2a**	Midwife toad *(Alytes obstetricans)*	BRIN-BD11 cells	High-fat-diet-induced obese Swiss mice	[85]
**Amolopin**	Frog *(Amolops loloensis)*	Rat INS-1 cells	ND	[86]
**Bombesin**	Frog (*Bombina bombina)*	HIT-T15 cells, isolated rat islets	Wild type baboon, wild type rats	[87,88,89,90,91,92]
**Brevinin-2-related peptide (B2RP**)	Mink frog *(Lithobates septentrionalis)*	BRIN-BD11 cells	HFD-induced obese Swiss mice	[93]
**Caerulein-related peptides**	Frog (*Xenopus borealis* and *Xenopus amieti)*	BRIN-BD11 cells	ND	[94]
**Crotamine**	Rattlesnake (*Crotalus durrisus terrificus)*	Isolated rat islets	ND	[95]
**Dermaseptin B-IV**	Frog *(Phyllomedusa trinitatis)*	BRIN-BD11 cells	ND	[96]
**Esculentin-2CHa**	Chiricahua leopard frog *(Lithobates chiricahuensis)*	BRIN-BD11 cells	Wild type and HFD-induced obese Swiss mice	[97,98]
**Hymenochirin-1b**	Frog Hymennochirus boettgeri	BRIN-BD11 cells	Wild type Swiss mice	[99,100]
**Magainin–AM1**	Volcano clawed frog *(Xenopus amieti)*	BRIN-BD11 cells	Wild type and HFD-induced obese Swiss mice	[101]
**Magainin–AM2**	Volcano clawed frog (*Xenopus amieti)*	BRIN-BD11 cells	Wild type and HFD-induced obese Swiss mice	[101]
**Melittin**	Honeybee *(Apis mellifera)*	Isolated mouse and rat islets	ND	[102,103]
**Ocellatin-L2**	Bullfrog *(Lithobates catesbeianus)*	BRIN-BD11 cells	ND	[104]
**Palustrin-2CBa**	Bullfrog *(Lithobates catesbeianus)*	BRIN-BD11 cells	ND	[105]
**Peptide Glycine-Leucine-Amide (PGLa)-AM1**	Frog *(Xenopus amieti)*	BRIN-BD11 cells and isolated mouse islets	ND	[106,107]
**Plasticin-L1**	Frog *(Leptodactylus laticeps)*	BRIN-BD11 cells	ND	[82]
**Pseudin-2**	frog (*Pseudis paradoxa)*	BRIN-BD11 cells	ND	[108]
**Ranatuerin-2CBd**	Bullfrog (*Lithobates catesbeianus*)	BRIN-BD11 cells	ND	[105]
**Temporin-1OE, -1Va, -1Vb** **1-Vc; -1DRb, -1TGb**	Frog (*Rana Ornativentris, Rana virgatipes, Rana draytonii, Rana Tagoi*)	BRIN-BD11 cells	ND	[109]
**Xenopsin and Xenopsin-AM2**	Frog (*Xenopus borealis* and *Xenopus amieti*)	BRIN-BD11 cells	ND	[94]

HFD: High Fat Diet; ND: Not Done.

**Table 4:** Polyphenols and alkaloids that stimulate insulin secretion in a mechanism that requires the closure of K^+^_ATP_ channels.

Compounds	Class	Group of the Active Substance	*In Vitro* Models	*In Vivo* Models	Reference(s)
**Astragalin**	Flavonol	Polyphenol	Isolated rat islets	ND	[140]
**Caffeine**	Purine	Alkaloid	ND	NMRI and BALB/c mice transplanted with mouse islets	[141,142,143]
**Ellagic acid**	Tannin	Polyphenol	Isolated mouse islets	ND	[144,145]
**Kaempferol**	Flavonol	Polyphenol	ND	ND	[146]
**Lupanine**	Quinolizidine	Alkaloid	Isolated rat islets	ND	[147]
**Nuciferin**	Aporphine	Alkaloid	Isolated mouse CD1 islets and INS-1 cells	ND	[148]
**Quercetin**	Flavonol	Polyphenol	Rat INS-1 cells	ND	[149]
**Resveratrol**	Stilbene	Polyphenol	MIN6 cells, HIT-T15, and RIN-m5F cells	Wistar Rats	[150]
**Schisandrol A, schisandrol B and schisandrin C**	Lignan	Polyphenol	Rat INS-1 cells	ND	[151]
**Sparteine**	Quinolizidine	Alkaloid	HIT-T15 cells	ND	[152]
**2R, 3R taxifolin 3-O-rhamnoside**	Flavanonol	Polyphenol	ND	Wild type mice	[153]
**Vindoline**	Indole	Alkaloid	Mouse MIN6 cells and isolated mouse islets	*db/db* mice and STZ/HFD-induced type 2 diabetic rats	[154]

ND: Not Done.

**Table 5:** Polyphenols and alkaloids that stimulate insulin secretion via a rise in cAMP levels.

Compounds	Subclass	Class of the Active Substance	*In Vitro* Models	*In Vivo* Models	Reference(s)
**Curcumin**	Curcuminoid	Polyphenol	Mouse MIN6 cells and isolated human islets	ND	[136]
**Daidzein**	Isoflavone	Polyphenol	Rat INS-1 cells and isolated mouse islets	ND	[155,156]
**Genistein**	Isoflavone	Polyphenol	Rat INS-1 cells, mouse MIN6 cells and isolated mouse islets, isolated human islets	Streptozotocin-induced diabetic mice	[137,157,158]
**Morphine**	Isoquinoline	Alkaloid	Isolated rat islets	ND	[139,159]
**Myricetin**	Flavonol	Polyphenol	Isolated rat islets	Wistar Rats	[160]
**Vanillic acid**	Benzoic acid	Polyphenol	Rat INS-1 cells and isolated rat islets	ND	[161]

ND: Not done.

**Table 6:** β-cell survival proteins induced by GLP-1RAs.

Protein Name	Protein Role	Reference(s)
AKT, also called PKB	AKT is a serine/threonine kinase which activates CREB, PDX1 and mammalian target of rapamycin (mTOR) complex 1. It inhibits glycogen synthase kinase 3 (GSK3β), caspase-9, FoxO1 and Bcl-2-associated death promoter (Bad)	[177,178]
MAK8IP1 also called Islet Brain 1/JIP1	Scaffold protein that tethers MAP3K/MAP2K/JNK.MAPK8IP1 is involved in the anti-apoptotic JNK signaling pathway	[179,180,181]
MAPK10/JNK3	Anti-apoptotic with unidentified targets. JNK3 is regulated by MAP8IP1/JIP-1/IB1	[176]
CREB	Transcription factor that positively regulates the expression of insulin receptor substrate 2, a key component of IGF-1 and insulin receptor signaling leading to AKT activation	[182]
ERK1/2	Ras-dependent extracellular signal-regulated kinase 1 (ERK1)/2 mitogen-activated protein (MAP) kinase pathway regulates cell survival	[183,184,185]
SERCA2b	P-type ATPase that regulates endoplasmic reticulum (ER) Ca^2+^ stores.	[186,187]
PDX-1	Transcription factor that determines endocrine cell fate and controls β-cell differentiation	[188]
PKA	Protein kinase A that phosphorylates transcription factor CREB	[14,185]
NKX6.1	Transcription factor that determines the specification of progenitor cells into mature functional β-cells. It maintains the function of adult pancreatic β-cells.	[189,190]
FoxO1	Forkhead transcription factor (Fox) of the O subclass. FoxO1 is a transcriptional effector of IGF signaling that controls β-cell mass through Pdx1	[191,192]
NRF2	The nuclear factor erythroid 2 (NFE2)-related factor 2 (Nrf2) is a leucine zipper (bZip) transcription factor that regulates oxidant levels	[193,194]
MAFA	While v-Maf musculoaponeurotic fibrosarcoma transcription factor A (MAFA) controls β-cell differentiation, it maintains the mature phenotype and viability of β-cells	[195]
XBP-1	X-box binding protein 1 (XBP1) is leucine zipper (bZIP) transcription factor that promotes ER biogenesis and activates the expression of ER chaperone genes	[196,197]
ERα	Estrogen receptor α (ERα) is a nuclear receptor that maintains the mitochondrial fission/fusion–mitophagy dynamics	[198,199]
Glucokinase	Transferase that phosphorylates glucose	[200]
PPARγ	Nuclear factor that regulates components of β-cell function and survival	[201,202]
AMPK	AMP-activated protein kinase that regulates β-cell survival via the mTOR pathway	[203,204]
Bcl2	Mitochondrial membrane protein that inhibits apoptosis	[205]

**Table 7:** Polyphenols and alkaloids targeting β-cell survival proteins.

Compounds	Class	Group of the Active Substance	Target Protein	*In Vitro* Models	*In Vivo* Models	Reference(s)
Curcumin	Curcuminoid	Polyphenol	AKT, FoxO1, SIRT1	Mouse MIN6 cells	ND	[222,223]
Epigallocatchin (EGCG)	Flavonol	Polyphenol	AKT, PDX1 FoxO1, Bcl2, AMPK	Rat RINm5F	ND	[224]
Anthocyanins	Anthocyanidins	Polyphenol	AMPK, Bcl2, PDX1	ND	KKAy diabetic mice STZ-induced diabetic rats	[225,226]
Dephinidin	Anthocyanidins	Polyphenol	AMPK	Rat RINm5F	ND	[227]
Cyanidin (Cyanidin-3-glucoside)	Anthocyanidins	Polyphenol	PPARγ, AKT, Bcl2	Mouse MIN6 and rat INS-1 cells	ND	[228,229,230]
Apigenin	Flavone	Polyphenol	AKT	Hamster HIT-T15 and rat RINm5F cells	ND	[231,232,233]
Luteolin	Flavone	Polyphenol	AKT, MAFA	Rat INS-1 cells, MIN6 cells and isolated mouse islets	Alloxan-induced diabetic rats	[234,235,236]
Kaempferol	Flavonol	Polyphenol	AKT, Bcl2, PKA, PDX1	Isolated human islets and INS-1 cells	ND	[146]
Quercetin	Flavonol	Polyphenol	ERK1/2, AKT	Rat RINm5F and INS-1 cells	ND	[232,237]
Myricetin	Flavonol	Polyphenol	PDX1, AKT	Rat RINm5F and INS-1 cells	ND	[238,239]
Naringenin	Flavonol	Polyphenol	AKT	INS-1 cells	ND	[237]
Genistein	Isoflavone	Polyphenol	ERK1/2, cAMP	Isolated human islets and INS-1 cells	STZ-induced diabetic rats	[240]
Silibinin	Flavinolignan	Polyphenol	PKA, PDX1	ND	STZ-induced diabetic rats	[241,242,243]
Resveratrol	Stilbene	Polyphenol	PDX1, FoxO1	Isolated human islets, isolated rat islets and INS-1 cells	HFD-induced diabetic mice	[244,245,246]
**Berberine**	Isoquinoline	Alkaloid	Nrf2	Rat INS-1 cells, MIN6 cells	ND	[131,219]

ND: Not Done, STZ: Streptozotocin.

## Data Availability

Not applicable.
